# Nervous Dyspepsia1Read at the thirty-second annual meeting of the Medical Association of Central New York, at Syracuse, October 17, 1899.

**Published:** 1899-12

**Authors:** B. C. Loveland

**Affiliations:** Syracuse, N. Y.


### NERVOUS DYSPEPSIA.[1]

BY B. C. LOVELAND, M. D., Syracuse, N. Y.

NERVOUS dyspepsia, otherwise called neurasthenia gastrica, is a functional neurosis, and may be considered as a distinct variety of neurasthenia. It is not the purpose of this paper to discuss neurasthenia of this or any other variety as an individual entity or disease, but to study this particular condition from the standpoint of practical experience.

Nervous dyspepsia is important, not only by reason of its frequency, but from the fact that it is so often intractable under the ordinary remedies. Many times it is not properly diagnosticated, and frequently it is mistaken for more serious disease. The causes that are responsible for nervous dyspepsia are, heredity or the nervous temperament, business worry, nervous shock, family cares, sedentary habits, insufficient muscular exercise, hastily eaten or improperly selected food, and an insufficient amount of water. In fact, any of the causes which so frequently lead up to other forms of neurasthenia. The symptoms commonly present are an unusual attention to the matter of digestion, a feeling of load or weight in the region

---

1. Read at the thirty-second annual meeting of the Medical Association of Central New York, at Syracuse, October 17, 1899.

of the stomach, eructation of gas, usually tasteless and often odorless. In the latter case the gas is only air which has been partially swallowed. and regurgitated in the effort to relieve the stomach of the feeling of weight. The patient also complains of slowness of digestion, and believes that one meal does not leave the stomach till the time for the next arrives. Beside the eructation of gas there is often eructation or regurgitation of food (not vomiting), the regurgitation occurring almost immediately after meals, and the food materials brought up being practically unchanged. The worst distress is generally complained of two or more hours after meals, and the patient also frequently says that as soon as he has begun to eat he feels full. Constipation, nervous and mental depression and insomnia are sometimes but symptoms of nervous dyspepsia. Such patients often come to us having cut off one thing after another in diet, and reduced the quantity of food until they are much emaciated, and they are fortunate if they have escaped the fad of abstaining from breakfast and similar absurdities, beside trying the numerous so-called dyspepsia cures so much advertised. For the more of such things they have tried the worse they are, and the more difficult it is to get them back into normal conditions and habits.

On physical examination such a patient will usually show a flabby skin, more or less sallow in color, a poorly developed muscular system, flaccid abdominal walls, and, by percussion, or auscultatory percussion, a dilated and displaced stomach will often be noted. And not infrequently a displaced kidney and displaced intestines may add to the picture. If the case is one of long standing much emaciation may, or may not, be present, depending on the degree to which the patient has restricted his diet. The appearance of the tongue is characteristic, but varied. It may be clean, red, with enlarged and congested papillæ on the tip; or a dull purplish color, thick and flabby, and marked with indentations by the teeth; or again, it may be "mapped," or coated white, with three or four spots of bright red, usually oval or circular in shape, situated on its anterior half. Any of these appearances may be present according to individual conditions or peculiarities. Repeatedly on making an examination of the stomach contents after a test meal they have been found normal in acidity and digestive elements, though occasionally absent, or marked reduction of the proportion of hydrochloric acid has been noted, as also hyperacidity. In other words, the contents of the stomach are usually found normal, though varying occasionally in the same individual, even at different times. From what has already been said it will be obvious that there are no pathological changes in the tissues of the stomach present in this disorder, and that it is a purely functional disease.

The rational treatment is, of course, to find the causes operative in each individual case and remove them if possible, and if this is impossible, contrive some means of obviating their effects. Counsel the patient to maintain a more serene state of mind, more freedom from worry and anxiety, to give more time to eating, and to preserve that time free from worrisome conversation, not to read the paper while eating; to use reasonable care in the selection of foods and once having eaten a meal to forget all about it, and occupy the mind with other things. Correct such bad habits as the regurgitation of food, or the eructation of gas, as in most instances it is unnecessary, and does not bring the hoped for relief. Tell the patient to eat what he can keep down, and keep down what he eats. Direct accurately the patient's dietary for the purpose in part of giving him less to think about and worry over. Few things that are wholesome for anyone to eat need be absolutely cut off if the patient is willing to obey orders thoroughly, but at first, for obvious reasons, it will not be best to give him too much liberty in choosing his variety. He may, however, be urged to eat as freely as his system may require, irrespective of his appetite, but when crowding the stomach in this manner a very simple diet must be used. Of course, a special diet list must be made for each individual, hence a general diet list in this paper would be impossible. It may be said, however, that sugar, sweets, pastries, fried dishes, and all but the plainest soups should be avoided, but can again be given, if the patient can be induced to make a radical change in his habits, like taking him from the office or professor's study and putting him into active work on a ranch, or its equivalent in such out-door exercise as horseback riding, walking, or cycling, three or four hours a day.

The results of any kind of out-door exercise will be better in proportion to the amount the patient takes (up to that just mentioned) and to the interest the patient takes in it. Hence the importance of prescribing an exercise that will be congenial to the patient, and for this reason the physician needs to know the advantages and disadvantages of the available out-door sports, for this class of patients often need to be convinced of the utility of such sport as well as the pleasurable quality of it. Where such a radical change in the habits and associations is impossible many things may be done to take the place of such change with varying degrees of benefit. Among these calisthenics, especially adapted to strengthen the muscles of the trunk and abdomen, hold an important place. Individual conditions would call for alterations, but a sample of such exercises would be as follows: flexion and extension of the arms and legs while standing, rising from a sitting posture (squatting down and rising up), bending forward and backward, bending sideways, first one side and then the other, lying on the back and elevating first one limb and then the other, and, finally, respiratory movements, deep and forcible. These last may often be taken best while lying on the back, as follows: hands down at sides, or clasped in front, as inhaling begins slowly raise hands, continuing until when the lungs are filled the hands touch the floor or couch on which the patient lies, as far as he can reach above or beyond his head. Return to former position while slowly exhaling. It is well to prescribe only about three such movements of a kind at the beginning, increasing as the strength increases, possibly adding one each day.

Faradic electricity is also of great value in restoring tone to the abdominal muscles, and I believe to the muscular structure of the stomach and intestines. If constipation exists, which cannot be overcome by the diet, exercise and electricity, phosphate of soda, grains 15, taken in a glass of hot water before each meal, will usually be found sufficient aid till the muscular activity of the alimentary tract has been restored. Nux vomica, in some form, is perhaps the remedy applicable to most cases, and the writer has used as frequently as any one prescription in this class of cases, the following: Pulv. rhubarb, soda bicarb., of each dr. ½, ext. nux. vom., gr. 6. M. div. in capsules, No. XXIV. S. Take one after each meal.

The results of the method of treatment described have been very satisfactory, and so far as it is possible to attribute results to any part of the management, it would seem that the success depended far more on the thoroughness with which the details of the patient's condition and habits of life were considered, or the careful physiological inventory taken, and the definite prescribing of new and better habits, than on the medicine given. For in some of the most emaciated and distressing cases no medicine has been given, simply diet and living in detail has been prescribed. Case reports might be added, but the paper has already reached the time allowed, and they will be omitted.

608 South Salina Street.

DISCUSSION.

Dr. Benedict, of Buffalo.—The doctor has given us a very interesting paper, and because it is interesting and good, it is possible to develop a little discussion on one or two points. I think that the only point that the doctor mentioned on which I radically disagree with him is on the use of soda. I cannot imagine any condition where we need to use nux vomica in which we would also want to use soda. Of course, there may be a case of superacidity in which we might need later to give soda to decrease the acidity, but in that case, we should have to be very careful to avoid any possible drug like strychnine that would increase the activity of the stomach. Of course, we all know physiologically that the stomach cannot digest until it is acid, and if the stomach is sour from organic sourness, it is no use giving soda, except as a very temporary relief.

There is one point—I think the doctor will agree with me here—and that is that the term "nervous dyspepsia" is a little bit misleading. It is something like "typhoid pneumonia." When we say "typhoid pneumonia," we are not quite sure whether we mean typhoid fever, complicated with pneumonia, or whether we mean a pneumonia that is in an extremely bad condition. So with the term, "nervous dyspepsia." Any dyspepsia is nervous; that is, any condition of the stomach that is not organic and hence is properly termed dyspepsia, and must be due to some nervous state. If we mean that the patient is nervous, I think we had better say that. You may say that is simply harping over a term, but there is this one practical point. I sometimes have patients referred to me with what might be termed "nervous dyspepsia." If I find that their stomach is normal in area, that there is no dilatation, that there is no special tendency toward vomiting, I say the less that patient knows about his stomach, the less he thinks about it, the less he has to do with me the better. Simply turn him or her back to the proper channel and have the treatment directed entirely away from the stomach. Quite recently, I have had two such cases referred to me, one a man, by a nervous specialist, the other a woman. In both cases, the patient was absolutely normal. I think it would be incorrect to call or treat such cases as nervous dyspepsia.

